# The Diversity of Spoon-Winged and Thread-Winged Lacewing Larvae Today and in Deep Time—An Expanded View

**DOI:** 10.3390/insects17010011

**Published:** 2025-12-20

**Authors:** Laura Buchner, Simon Linhart, Gideon T. Haug, Florian Braig, Thomas Weiterschan, Patrick Müller, Joachim T. Haug, Carolin Haug

**Affiliations:** 1 Biocenter, Ludwig-Maximilians-Universität München, Großhaderner Str. 2, 82152 Planegg-Martinsried, Germany; laura.buchner@campus.lmu.de (L.B.);; 2 Fakultät für Biowissenschaften, Universität Heidelberg, Im Neuenheimer Feld 234, 69120 Heidelberg, Germany; 3 Ecology and Genetics Research Unit, University of Oulu, Pentti Kaiteran Katu 1, 90570 Oulu, Finland; 4 Independent Researcher, 64739 Höchst im Odenwald, Germany; 5 Independent Researcher, 66482 Zweibrücken, Germany; 6 GeoBio-Center at LMU, Richard-Wagner-Str. 10, 80333 München, Germany

**Keywords:** Neuroptera, Nemopteridae, Nemopterinae, Crocinae, Burmese amber, convergent evolution

## Abstract

Larvae of lacewings (Neuroptera) are (mostly) ferocious predators, and the most well known are the larvae of antlions. In the larger group of antlion-like lacewings, the larvae resemble those of antlions to a certain degree but have certain peculiarities. Larvae of the group Nemopteridae can be easily separated into two groups: the larvae of Crocinae (thread-winged lacewings), also known as long-necked antlions, have relatively slender mouthparts and long necks, as the name suggests; the larvae of Nemopterinae (spoon-winged lacewings) have stout mouthparts and bodies. We here report new lacewing larvae of the group Nemopteridae from about 100-million-year-old Kachin amber, Myanmar. The new findings significantly expand the dataset of these animals in the fossil record. Quantitative analyses comparing the fossil and extant larvae support earlier indications that the morphological diversity of the fossils was in fact larger, yet the results are not as conclusive as in other ingroups of Neuroptera. The comparison also indicates that convergent evolution played an important role in the evolution of larvae of antlion-like lacewings.

## Simple Summary

Most larvae of lacewings (Neuroptera) prey on other animals, such as the larvae of antlions. In the larger group of antlion-like lacewings, the larvae look similar to those of antlions but have their own certain characteristics. Larvae of the group Nemopteridae can be easily separated into two groups: the larvae of thread-winged lacewings (Crocinae), also known as long-necked antlions, have relatively slender mouthparts and long necks, as the name suggests; the larvae of spoon-winged lacewings (Nemopterinae) have stout mouthparts and bodies. We here report new lacewing larvae of the group Nemopteridae from about 100-million-year-old Kachin amber, Myanmar. With the new findings several new fossil larvae of this group are now known. We used shape analyses to compare the fossil and modern larvae. Our results support those of earlier studies, more exactly that the morphological diversity of the fossils was larger than of the modern larvae. However, the results are not as conclusive as in other lacewing groups. The comparison also indicates that independent evolution of similar morphologies played an important role in the evolution of larvae of antlion-like lacewings.

## 1. Introduction

Biodiversity is declining in recent times [1,2,3]. Human-induced climate change, loss of habitats, and general human interference together have caused an enormous loss in biodiversity in the last 50 years [4]. To enhance our understanding of the processes of the current diversity loss, we can look at comparable declines in the past.

In the modern fauna, a major share of the animal biodiversity in continental ecosystems is that of Insecta, or its ingroup Holometabola [5]. The four most species-rich lineages of this group, Coleoptera (beetles) [6,7,8,9,10], Diptera (true flies) [11,12,13], Hymenoptera (wasps) [14,15,16], and Lepidoptera (moths) [17,18] are considered as “the big four”. Each of these includes more than 100,000 formally described species [5,19,20]. In contrast, Neuroptera (lacewings) has only about 6000 species in the extant fauna; yet, it has been considered to have been more diverse in the past [21,22] and is therefore especially interesting for studying processes of loss of diversity. Quantitative morphological studies have demonstrated that the loss expressed by Neuroptera is mostly found in its larvae [23].

Lacewing larvae can be easily recognised by their prominent stylets, forward-projecting mouthparts for piercing and sucking. They are formed by the mandible and maxilla on each side and form a central channel which the larvae use to inject saliva and venom into the prey before sucking out the pre-digested and liquefied tissues [24,25,26].

Nemopteridae is an ingroup of Myrmeleontiformia, the antlion-like lacewings. Nemopteridae has two ingroups [27]: Nemopterinae (spoon-winged lacewings) and Crocinae (thread-winged lacewings). Adults of both groups have spectacular specialised hindwings (cf. names), yet the larvae of both are also spectacular in certain ways. Many larvae of Nemopterinae are deep-digging predators with broad heads and short, massive stylets [28]. Many larvae of Crocinae have long neck regions giving them quite a peculiar appearance [29].

Few fossil larvae have been reported which share certain aspects with the modern larvae of Nemopteridae [30,31] but also differ in morphological, and therefore likely ecological, aspects [32]. Only few fossils have been available, which did not provide a clear signal of a possible loss of diversity for Nemopteridae [33,34]. However, larger-scaled, sample-size-corrected analyses have provided such a signal [23].

Here we report new fossils, possibly representing larvae of Nemopteridae, from about 100 million years old Kachin amber, Myanmar. We use these as a basis for an expanded quantitative morphological comparison of the diversity of larvae of Nemopteridae over time.

## 2. Material and Methods

### 2.1. Material

All 17 new specimens are preserved in approximately 100 million years old Kachin amber, Myanmar, and come from three different collections. Thirteen specimens are deposited in the Palaeo-Evo-Devo Research Group Collection of Arthropods, Ludwig-Maximilians-Universität München, Germany, under repository numbers PED 0515, 0915, 1408, 1422, 1812, 1889, 2106, 2400, 2407, 2423, 2493, 2504, and 2712. The amber pieces were legally purchased via the trading platform eBay.com from various traders (burmite-miner, burmitefossil, macro-cretaceous). Two specimens come from the collection of one of the authors (TW; BuB 14 and BuB 15). Two additional specimens come from the collection of one of the other authors (PM; BUB 0723, BUB 3382b). As PED 0515, PED 0915, and PED 2423 are rather poorly preserved, these specimens are only presented as Supplementary (Figure S1).

Literature data on larvae of the group Nemopteridae came from various sources:

- Extant larvae of the group Crocinae [21,24,25,27,29,33,35,36,37,38,39,40,41,42,43,44,45,46,47,48,49,50,51,52,53,54,55,56,57,58,59];
- Extant larvae of the group Nemopterinae [27,28,60,61,62,63,64,65,66,67];
- Fossil larvae of the group Nemopteridae [30,31,32,33,34].

### 2.2. Imaging and Documentation

The amber pieces were documented with a Keyence VHX-6000 digital microscope (Keyence, Osaka, Japan). A panorama function was used for combining different adjacent frames. High-resolution images are produced by focusing through the different focus layers in each frame. Additionally, different illumination settings were used: ring illumination, cross-polarised coaxial light, or transmitted light. The first two settings were performed in front of a black and a white background. The transmitted light was performed with a glass plate. The specimens were usually documented from both sides, if accessible. Only the best images were further processed. Adobe Photoshop (different versions; https://www.adobe.com/) was used for illustration.

### 2.3. Measurements

For the measurements performed on the new specimens, the open-source software Fiji 2.0.0 (https://fiji.sc/) or ImageJ 1.53 (https://imagej.net/) [68] was used. The total body length of the larvae (including the stylets) or the accessible body length was measured. If the specimen was bent, the measurement was performed along the body axis, following the curvature.

### 2.4. Outlines

Ten combinations of different body parts were used to perform analyses. Stylet, head capsule, prothorax, and the trunk without prothorax were outlined. The combinations used were stylet, body with stylet, body without stylet, body without head, body without head and prothorax, head with stylet, head without stylet, prothorax with head and stylet, prothorax with head, and prothorax. Body appendages were not included in the outlines. Outlines were reconstructed in dorsal view. If the specimen was bent, it was artificially straightened in the vector graphics program used for drawing the outlines [69]. The outlines were drawn in Adobe Illustrator (different versions; https://www.adobe.com/) and Inkscape (https://inkscape.org/) and combined with outlines from earlier studies (Table S1). The enumeration of the specimens documented in this study continues from the dataset of our previous studies [23,33,34].

### 2.5. Shape Analysis

Ten Elliptic Fourier analyses were performed. Nine analyses were performed in SHAPE (http://lbm.ab.a.u-tokyo.ac.jp/~iwata/shape/index.html) following the method of Iwata and Ukai [70] and Braig et al. [71]. One dataset (prothorax) had difficulties with the alignment in SHAPE and was therefore analysed in the R statistics environment (ver. 4.1.0) [72] using the package Momocs (https://momx.github.io/Momocs/) [73]. The two principal components (PC) explaining most of the variance were plotted against each other in scatterplots using OpenOffice Calculator 4.1.15 (https://www.openoffice.org/) or the R statistics environment. The plots were redrawn using Adobe Photoshop (different versions).

## 3. Results

### 3.1. Descriptions of New Fossil Larvae: Possible Larvae of Crocinae

**Figure 1:**
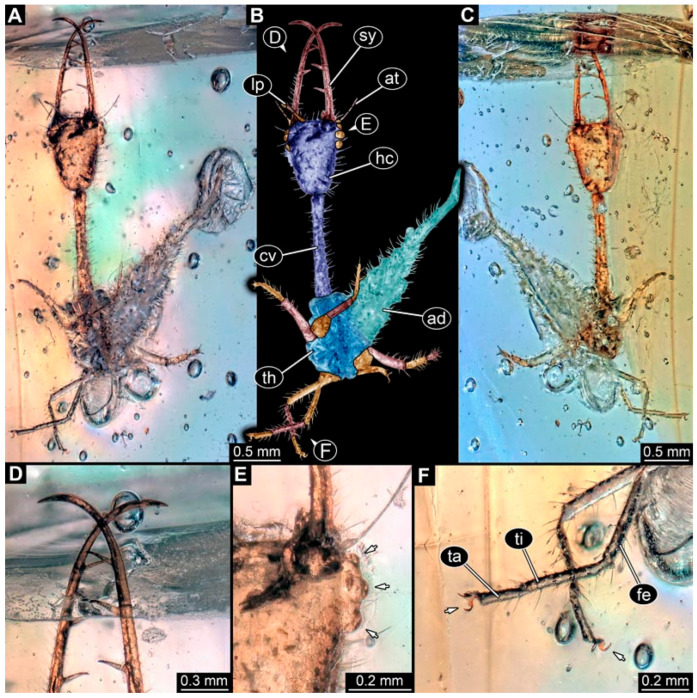
Possible larva of Crocinae, specimen 0471 (BUB 0723). (**A**) Ventral view. (**B**) Ventral view, colour-marked. (**C**) Dorsal view. (**D**) Close-up of stylet with teeth in ventral view. (**E**) Close-up of head capsule in ventral view; arrows mark stemmata. (**F**) Close-up of locomotory appendages (legs) in ventral view; arrows mark claws. Abbreviations: ad = abdomen; at = antenna; cv = cervix (sclerotised part of the neck); fe = femur; hc = head capsule; lp = labial palp; sy = stylet; ta = tarsus; th = thorax; ti = tibia.

**Figure 2:**
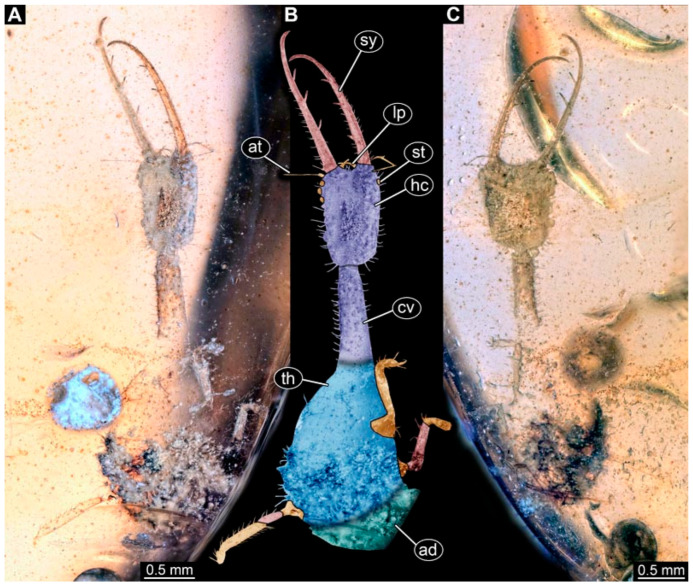
Possible larva of Crocinae, specimen 0474 (PED 2106). (**A**) Ventral view. (**B**) Ventral view, colour-marked. (**C**) Dorsal view. Abbreviations: ad = abdomen; at = antenna; cv = cervix (sclerotised part of neck); hc = head capsule; lp = labial palp; st = stemmata; sy = stylet; th = thorax.

**Figure 3:**
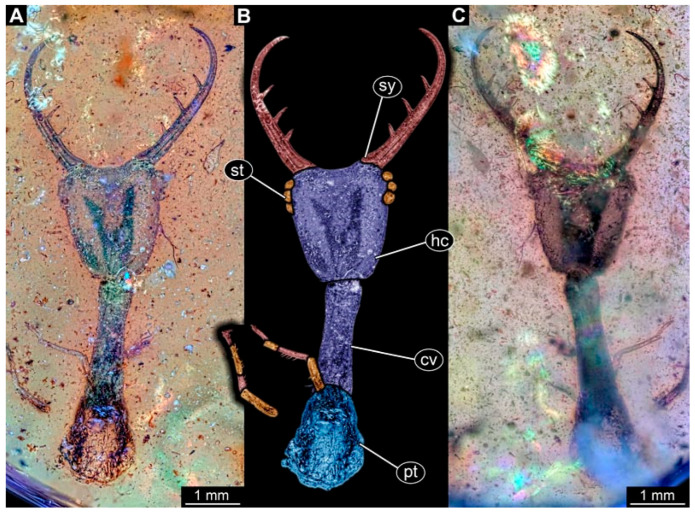
Possible larva of Crocinae, specimen 0475 (PED 2400). (**A**) Ventral view. (**B**) Ventral view, colour-marked. (**C**) Dorsal view. Abbreviations: cv = cervix (sclerotised part of neck); hc = head capsule; pt = prothorax; st = stemmata; sy = stylet.

**Figure 4:**
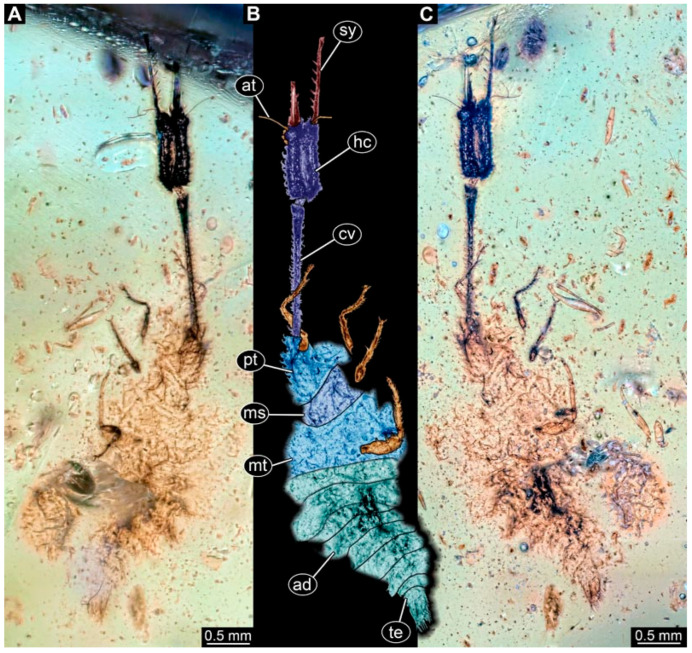
Possible larva of Crocinae, specimen 0476 (PED 2407). (**A**) Dorsal view. (**B**) Dorsal view, colour-marked. (**C**) Ventral view. Abbreviations: ad = abdomen; at = antenna; cv = cervix (sclerotised part of neck); hc = head capsule; ms = mesothorax; mt = metathorax; pt = prothorax; sy = stylet; te = trunk end.

**Figure 5:**
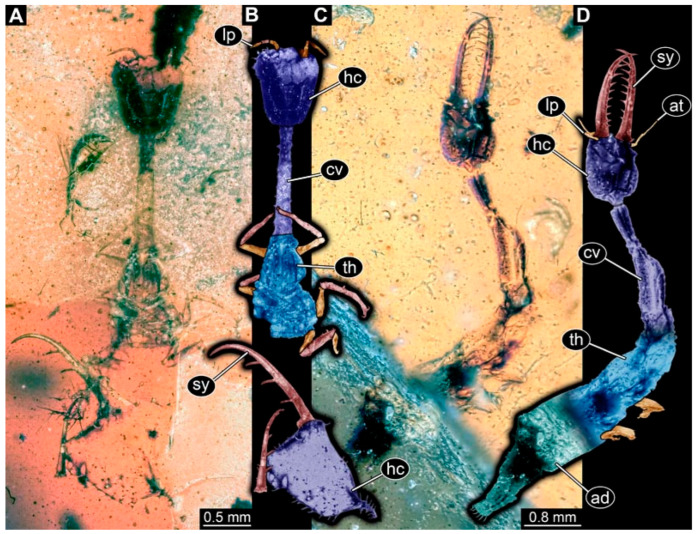
Possible larvae of Crocinae. (**A**,**B**) Specimen 0478 (PED 2493). (**A**) Dorsal view. (**B**) Dorsal view, colour-marked. (**C**,**D**) Specimen 0479 (PED 2504). (**C**) Dorsal view. (**D**) Dorsal view, colour-marked. Abbreviations: ad = abdomen; at = antenna; cv = cervix (sclerotised part of neck); hc = head capsule; lp = labial palp; sy = stylet; th = thorax.

**Figure 6:**
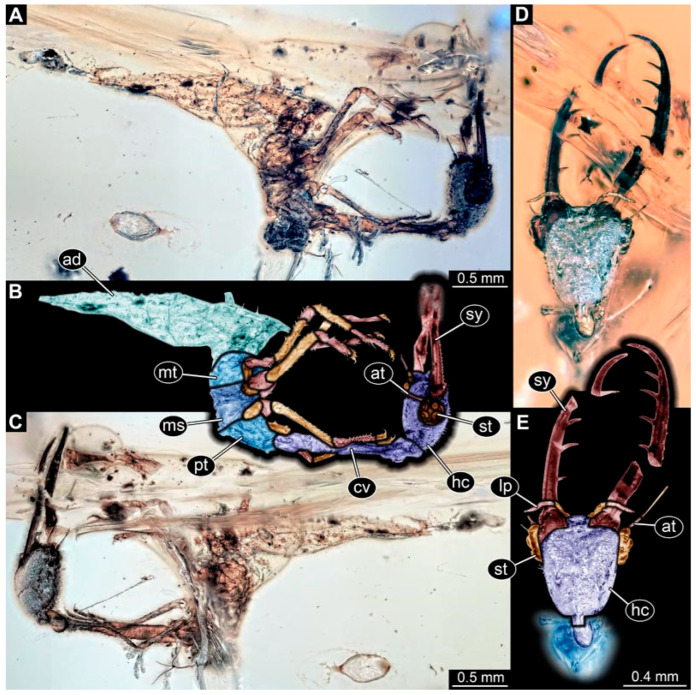
Possible larva of Crocinae, specimen 0480 (PED 2712). (**A**) Lateral view. (**B**) Lateral view, colour-marked. (**C**) Lateral view, other side. (**D**) Head in ventral view. (**E**) Head in ventral view, colour-marked. Abbreviations: ad = abdomen; at = antenna; cv = cervix (sclerotised part of neck); hc = head capsule; lp = labial palp; ms = mesothorax; mt = metathorax; pt = prothorax; st = stemmata; sy = stylet.

**Figure 7:**
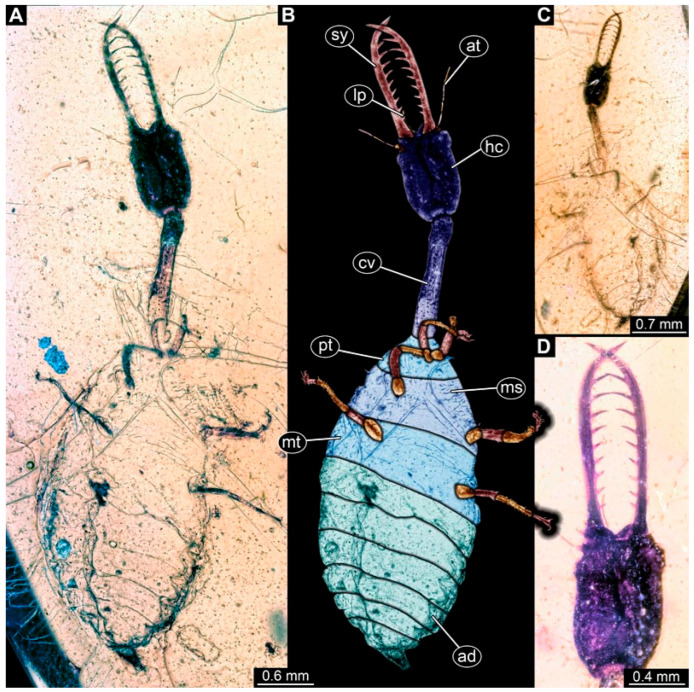
Possible larva of Crocinae, specimen 0481 (BuB 15). (**A**) Ventral view. (**B**) Ventral view, colour-marked. (**C**) Dorsal view. (**D**) Head in ventral view. Abbreviations: ad = abdomen; at = antenna; cv = cervix (sclerotised part of neck); hc = head capsule; lp = labial palp; ms = mesothorax; mt = metathorax; pt = prothorax; sy = stylet.

**Figure 8:**
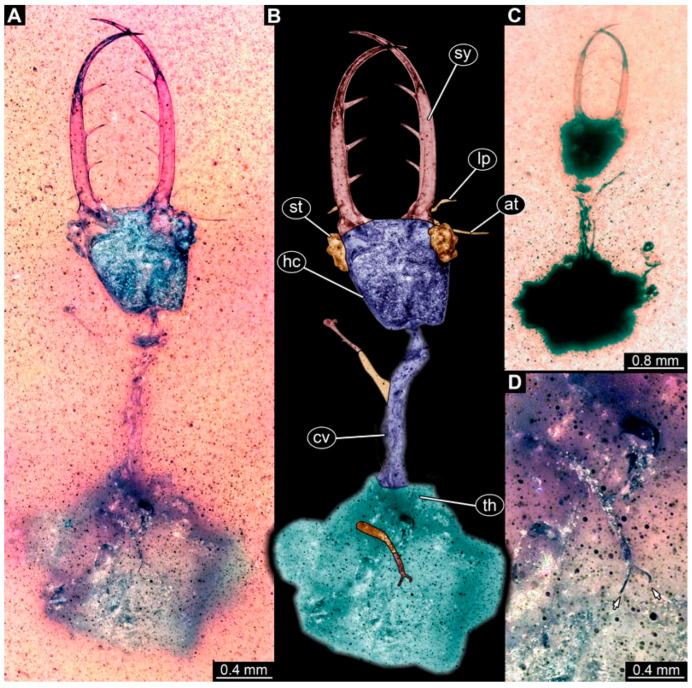
Possible larva of Crocinae, specimen 0482 (BuB 14). (**A**) Ventral view. (**B**) Ventral view, colour-marked. (**C**) Dorsal view. (**D**) Close-up of locomotory appendages, arrows mark claws. Abbreviations: at = antenna; cv = cervix (sclerotised part of neck); hc = head capsule; lp = labial palp; st = stemmata; sy = stylet; th = thorax.

(1) Specimen 0471 (BUB 0723) is accessible in ventral (Figure 1A,B) and dorsal (Figure 1C) view. The stylets bear three teeth each (Figure 1D). The eyes are prominent on both sides of the head (Figure 1E). The sclerotised part of the neck (cervix) is elongated and seems to be rather narrow. Claws are visible distally on locomotory appendages (Figure 1F). The specimen has a length of about 6.7 mm.

(2) Specimen 0474 (PED 2106) is well accessible in ventral (Figure 2A,B) and dorsal view (Figure 2C). Each stylet bears three teeth. The cervix is elongated, rather broad, and relatively short. The posterior trunk seems to be missing. The preserved part of the specimen has a length of about 5.6 mm.

(3) Specimen 0475 (PED 2400) is well accessible in ventral (Figure 3A,B) and dorsal view (Figure 3C). Each stylet bears three teeth. The cervix is rather broad and relatively short. The posterior trunk seems to be missing. The preserved part of the specimen has a length of about 8.4 mm.

(4) Specimen 0476 (PED 2407) is well accessible in dorsal (Figure 4A,B) and ventral view (Figure 4C). The distal ends of the stylets seem to be missing. Each stylet seems to bear multiple teeth. The cervix is long in comparison to other specimens and narrow. The specimen has a length of about 7.3 mm. This specimen was not included in the final analysis.

(5) Specimen 0478 (PED 2493) is well accessible in dorsal view (Figure 5A,B) but partly concealed by dirt. The stylet including one half of the head capsule (presumably the dorsal one) seems to be separated from the rest of the body but is still present. The stylet bears three teeth each. The cervix is elongated and rather broad. The preserved part of the specimen has a length of about 4.6 mm, including the 1.3 mm-long stylets.

(6) Specimen 0479 (PED 2504) is well accessible in dorsal view (Figure 5C,D) but partly concealed by disturbances in the amber. The stylet bears nine teeth each. The cervix is elongated and broad, slightly widening anteriorly. The specimen has a length of about 7.8 mm.

(7) Specimen 0480 (PED 2712) is accessible in lateral (Figure 6A–C) view but partly concealed by disturbances in the amber. The head is also accessible in ventral view (Figure 6D,E). Each stylet bears four teeth. Eyes are very prominent. The cervix seems to be rather long and narrow. Each locomotory appendage bears claws. The specimen has a length of about 5.4 mm.

(8) Specimen 0481 (BuB 15) is well accessible in ventral (Figure 7A,B) and also accessible in dorsal view (Figure 7C). The stylets bear nine teeth each (Figure 7D). The cervix is long and narrow. The specimen has a length of about 6.9 mm.

(9) Specimen 0482 (BuB 14) is well accessible in ventral (Figure 8A,B,D) and dorsal view (Figure 8C) but partly concealed by milky coating (Verlumung) in ventral and dorsal view. The posterior trunk seems to be missing. The stylets bear three teeth each. The cervix appears long and rather narrow. The trunk seems to be missing. The preserved part of the specimen has a length of about 5.0 mm.

### 3.2. Descriptions of New Fossil Larvae: Possible Larvae of Nemopterinae

**Figure 9:**
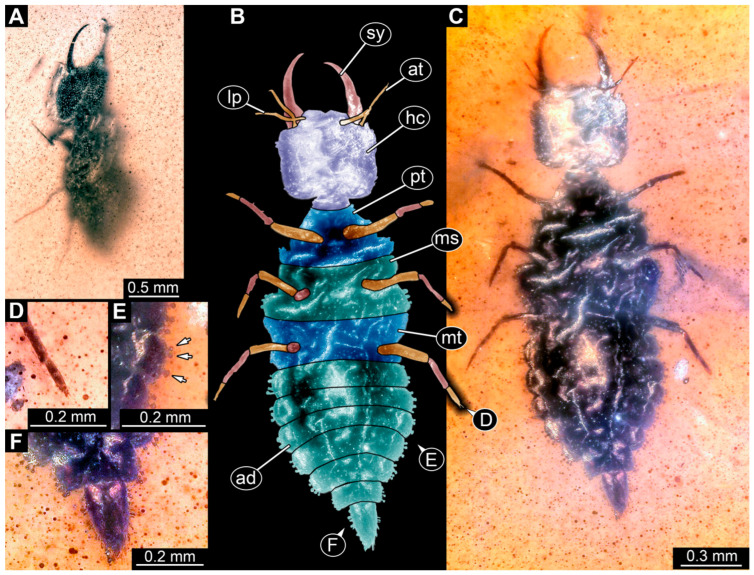
Possible larvae of Nemopterinae. (**A**) Specimen 0630 (PED 1408), dorsal view. (**B**–**F**) Specimen 0632 (PED 1812). (**B**) Ventral view, colour-marked. (**C**) Ventral view. (**D**) Close-up of locomotory appendage. (**E**) Close-up of abdomen in ventral view, arrows mark hammer-headed setae. (**F**) Close-up of trunk end in ventral view. Abbreviations: ad = abdomen; at = antenna; hc = head capsule; lp = labial palp; ms = mesothorax; mt = metathorax; pt = prothorax; sy = stylet.

**Figure 10:**
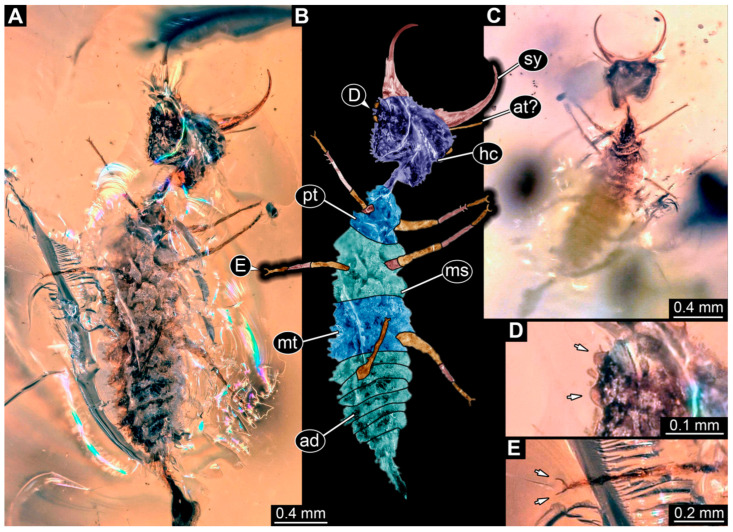
Possible larva of Nemopterinae, specimen 0631 (PED 1422). (**A**) Ventral view. (**B**) Ventral view, colour-marked. (**C**) Dorsal view. (**D**) Close-up of head in ventral view, arrows mark stemmata. (**E**) Close-up of locomotory appendage in ventral view, arrows mark claws Abbreviations: ad = abdomen; at? = possible antenna; hc = head capsule; ms = mesothorax; mt = metathorax; pt = prothorax; sy = stylet.

**Figure 11:**
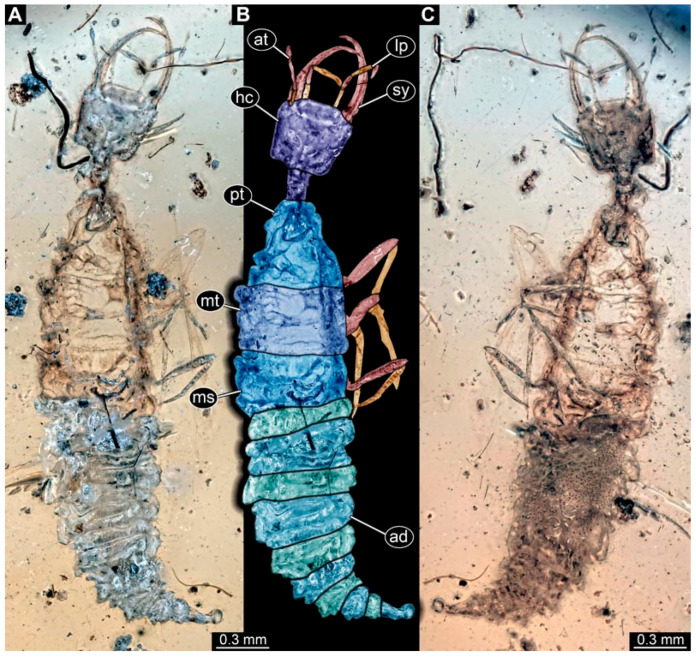
Possible larva of Nemopterinae, specimen 0633 (PED 1889). (**A**) Dorsal view. (**B**) Dorsal view, colour-marked. (**C**) Ventral view. Abbreviations: ad = abdomen; at = antenna; hc = head capsule; lp = labial palp; ms = mesothorax; mt = metathorax; pt = prothorax; sy = stylet.

**Figure 12:**
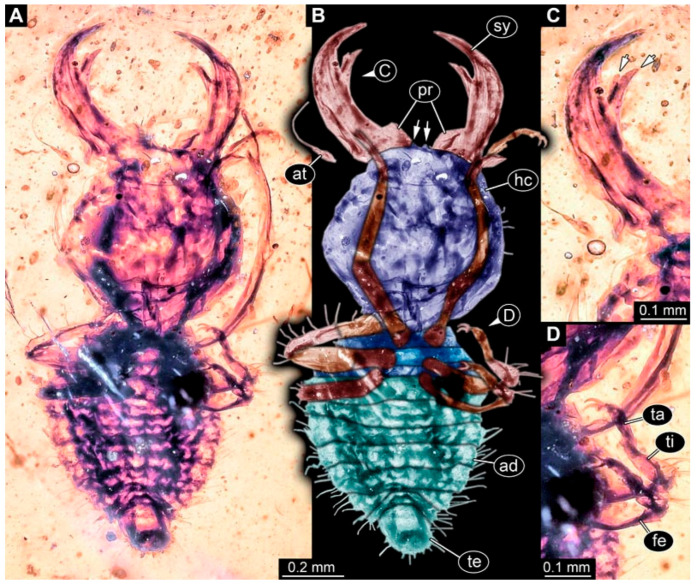
Possible larva of Nemopterinae, specimen 0901 (BUB 3382b). (**A**) Ventral view. (**B**) Ventral view, colour-marked, arrows mark small protrusions on head capsule. (**C**) Close-up of stylet with prominent tooth, arrows mark cusps. (**D**) Close-up of locomotory appendage. Abbreviations: ad = abdomen; at = antenna; fe = femur; hc = head capsule; pr = protrusion; sy = stylet; ta = tarsus; te = trunk end; ti = tibia.

(10) Specimen 0630 (PED 1408) is largely concealed by milky coating (Verlumung) in slightly oblique dorsal view (Figure 9A), and only the rough outline is apparent. The specimen has a length of about 2.4 mm.

(11) Specimen 0631 (PED 1422) is well accessible in ventral (Figure 10A,B) and dorsal view (Figure 10C). The head is rather round. The cervix seems to be rather long in comparison to other specimens of Nemopterinae. Eyes are well accessible (Figure 10D). Each distal end of the walking appendages bears claws (Figure 10E). The specimen has a length of about 2.8 mm.

(12) Specimen 0632 (PED 1812) is well accessible in ventral view (Figure 9B,C). The head is rather square-shaped in ventral view. Each distal end of the walking appendages bears claws (Figure 9D). The setae are hammer headed (Figure 9E). The trunk end is broad spearhead-shaped (Figure 9F). The specimen has a length of about 2.4 mm.

(13) Specimen 0633 (PED 1889) is well accessible in dorsal (Figure 11A,B) and ventral view (Figure 11C). The head is square shaped in dorsal and ventral view. The cervix seems to be rather long in comparison to other specimens of Nemopterinae. The specimen has a length of about 3.8 mm.

(14) Specimen 0901 (BUB 3382b) is well accessible in ventral view (Figure 12A,B). The head is rather circular. Stylets are prominent but stout, bearing a single tooth each. Each stylet proximally contains a broad protrusion. A tooth with two cusps, arranged abaxially, can be seen (Figure 12C). The trunk is rather small in comparison to the head, indicating an early developmental state. Locomotory appendages are well preserved distally with a pair of claws (Figure 12D).

### 3.3. Shape Analysis

The details of all shape analyses are available in the Supplementary (Files S1 and S2).

*Analysis (1) Head and stylets:* The shape analysis of the head capsule with stylets resulted in six effective principal components, summarising a total of 91.3% of the overall variation in the dataset. The first two principal components sum up to 75.9% of the overall variation in the dataset.

PC1 explains 54.4% of the overall variation. It describes round to rectangular head shapes with broad and short to narrow and long stylets. Low values indicate a round head with a broad anterior rim with short, broad stylets and tapering stylet tips. High values indicate a rectangular head with long, strongly curved stylets.

PC2 explains 21.5% of the overall variation. It is dominated by the shape of the head and the length of the stylets. It describes round to triangular head shapes with narrow to broad stylets. Low values indicate a broad, round head being narrower anteriorly with narrow stylets that are in the distal region as broad as in the proximal region. High values indicate a rather triangular head being narrower posteriorly with stout distally tapering stylets.

*Analysis (2) Stylets:* The shape analysis of the stylets resulted in five effective principal components, summarising a total of 91.5% of the overall variation in the dataset. The first two principal components sum up to 75.2% of the overall variation in the dataset.

PC1 explains 66.6% of the overall variation. It is dominated by the shape and the curving of the stylets. It describes slender and curved to stout and straight stylets. Low values indicate slender stylets with long, strongly curved stylet tips. High values indicate straight, stout stylets with teeth.

PC2 explains 8.6% of the overall variation. Low values indicate rather straight, tapering stylets. High values indicate long, curved, tapering stylets.

*Analysis (3) Head capsule:* The shape analysis of the head capsule resulted in seven effective principal components, summarising a total of 90.8% of the overall variation in the dataset. The first two principal components sum up to 55.8% of the overall variation in the dataset.

PC1 explains 28.8% of the overall variation. It is dominated by the shape of the anterior and posterior rim. It describes convex to concave rims. Low values indicate a convex anterior edge of the head capsule and a convex posterior rim. High values indicate a concave anterior edge of the head capsule with a concave posterior rim.

PC2 explains 27.0% of the overall variation. It describes trapezoidal to round head shapes. Low values indicate a rather trapezoidal head being narrower posteriorly. High values indicate a round head with a concave posterior rim.

*Analysis (4) Body including stylets:* The shape analysis of the body outline including head capsule and stylets resulted in seven effective principal components, summarising a total of 92.9% of the overall variation in the dataset. The first two principal components sum up to 69.6% of the overall variation in the dataset.

PC1 explains 46.3% of the overall variation. It is dominated by the length of the cervix, yet the shape of the stylets also influences this PC. It describes a long to short cervix with long to short stylets. Low values indicate a rather short trunk with a long cervix with long, slender stylets. High values indicate a rather long trunk with a short cervix with short, stout stylets.

PC2 explains 23.3% of the overall variation. It is dominated by the shape of the head. It describes a short to long cervix and a large to small head capsule. Low values indicate a concave lateral rim of the head, a short cervix, and a large head capsule. High values indicate straight lateral rims of the head, a long cervix, and a small head capsule.

*Analysis (5) Body outline without stylets:* The shape analysis of the entire body with head capsule but without stylets resulted in six effective principal components, summarising a total of 92.3% of the overall variation in the dataset. The first two principal components sum up to 73.6% of the overall variation in the dataset.

PC1 explains 54.4% of the overall variation. It is dominated by the shape of the head. It describes narrow heads with a long cervix to broad heads with a short cervix, yet also the shape of the posterior end of the body seems to influence this PC. Low values indicate a rounded trunk with a narrow head and a long cervix. High values indicate an elongated tapering posterior rim of the body with a short, slender cervix and a broad, round head.

PC2 explains 19.3% of the overall variation. It is dominated by the shape of the body and the head capsule. It describes straight to S-shaped lateral body rims and small to large head capsules. Low values indicate straight lateral body rims and a small, round head. High values indicate an S-shaped lateral body rim with a round trunk and an elongated head capsule.

*Analysis (6) Head, stylets, and prothorax:* The shape analysis of the head with stylets and prothorax resulted in seven effective principal components, summarising a total of 92.3% of the overall variation in the dataset. The first two principal components sum up to 68.3% of the overall variation in the dataset.

PC1 explains 40.5% of the overall variation. It is dominated by the shape of the stylet tips. It describes long, massive to short, tapering stylet tips, yet also the shape of prothorax with cervix seems to influence this PC. Low values indicate a concave lateral rim of prothorax with cervix and long massive stylet tips that are in the distal region as broad as in the proximal region. High values indicate a straight, narrow prothorax with cervix and rather short, tapering stylets.

PC2 explains 27.8% of the overall variation. It is dominated by the size of the prothorax and the shape of the stylets. It describes a small to large prothorax and short, straight to long, curved stylets. Low values indicate a small prothorax and short, straight stylets. High values indicate a large, rather concave lateral rim of the prothorax and long, tapering, strongly curved stylets.

*Analysis (7) Head and prothorax (no stylets):* The shape analysis of the head capsule without stylets but with prothorax resulted in six effective principal components, summarising a total of 92.4% of the overall variation in the dataset. The first two principal components sum up to 76.2% of the overall variation in the dataset.

PC1 explains 54.1% of the overall variation. It is dominated by the shape of the posterior rim of the prothorax and the elongation of the head capsule. It describes rounded posterior rims and short head capsules to rectangular posterior edges and long head capsules. Low values indicate a narrow prothorax with a rounded posterior rim and a short head capsule with a long cervix. High values indicate a broad prothorax with a rectangular posterior edge and a long head capsule with a short cervix.

PC2 explains 22.0% of the overall variation. It is dominated by the shape of the cervix. It describes straight to concave lateral rims of the cervix. Low values indicate a straight, narrow cervix. High values indicate a concave lateral rim of the cervix with a narrow posterior rim and a broad anterior rim.

*Analysis (8) Prothorax:* The shape analysis of the prothorax resulted in six effective principal components, summarising a total of 98.4% of the overall variation in the dataset. The first two principal components sum up to 92.7% of the overall variation in the dataset.

PC1 explains 85.4% of the overall variation. It is dominated by the width of the prothorax. It describes a narrow to broad prothorax, yet also the anterior and posterior ends seem to influence this PC. Low values indicate a relatively narrow prothorax with distinctive elongated anterior and posterior ends. High values indicate a broad prothorax with an anterior end that is slightly narrower than the rest of the prothorax and a straight posterior rim.

PC2 explains 7.3% of the overall variation. It is dominated by the position of the narrowest part of the prothorax and the shape of the posterior end. Low values indicate a further posteriorly located narrowest part of the prothorax and a small round posterior end. High values indicate a further anteriorly located narrowest part of the prothorax and a broad posterior end.

*Analysis (9) Body outline without head capsule, *i.e.,* trunk:* The shape analysis of the body without head capsule resulted in seven effective principal components, summarising a total of 93.0% of the overall variation in the dataset. The first two principal components sum up to 74.6%. of the overall variation in the dataset.

PC1 explains 55.4% of the overall variation. It is dominated by the shape of the thorax. It describes concave to convex lateral rims of the thorax, yet also the shape of the anterior rim of the body seems to influence this PC. Low values indicate a relatively convex lateral rim of the thorax with a fluent transition between thorax and posterior trunk and a tapering anterior rim of the body. High values indicate a relatively concave lateral rim of the thorax with a clear distinction from the posterior trunk and a rounded anterior rim of the body.

PC2 explains 19.2% of the overall variation. It is dominated by the shape of the thorax and the posterior end of the body. Low values indicate a rather convex lateral rim of the thorax with a fluent transition between thorax and posterior trunk and a rounded posterior end of the body. High values indicate a rather convex lateral rim of the thorax with a clear distinction from the posterior trunk and a tapering posterior end of the body.

*Analysis (10) Trunk outline without prothorax:* The shape analysis of the body without head capsule resulted in seven effective principal components, summarising a total of 90.3% of the overall variation in the dataset. The first two principal components sum up to 48.7% of the overall variation in the dataset.

PC1 explains 28.2% of the overall variation. It is dominated by the shape of the posterior end and anterior rim of the mesothorax. Low values indicate a slightly tapering, elongated posterior end and a rounded anterior rim of the mesothorax. High values indicate a narrow tapering posterior end and a concave anterior rim.

PC2 explains 20.5% of the overall variation. It is dominated by the shape of the posterior end of the body. Low values indicate a slightly concave posterior rim. High values indicate a strongly concave posterior rim, resulting in a narrow trunk end.

## 4. Discussion

### 4.1. Identity of the Long-Necked Specimens (0471–0482)

The presence of stylets, the overall arrangement of the head structures, and the distinct sclerotized cervix (neck) between head and prothorax clearly identify all reported specimens as larvae of Neuroptera [74]. Within Neuroptera further identification is based on prominent features. Extant larvae of Crocinae are rather easy to identify since they are quite unique in their appearance compared to other larvae of Neuroptera. They have long and slender stylets and squared to triangular head capsules. However, the most obvious characteristic is their extremely elongated cervix [21]. Especially the species groups *Dielocroce* and *Necrophylus* (unclear if synonymous) stand out, not only with their extraordinary length of the cervix [29] but also with their triangular head and relatively smooth mandibles lacking teeth [75]. Especially the long cervix is well apparent in many of the new specimens (Figure 1, Figure 2, Figure 3, Figure 4, Figure 5, Figure 6, Figure 7 and Figure 8), yet other aspects of the new larvae differ.

Many depicted extant larvae of Crocinae usually do not show teeth [33] or sometimes have small ones, yet most of the depicted extant specimens are in the later larval stages. Stage 1 larvae of Crocinae usually seem to possess distinct teeth (three large ones, sometimes additional smaller ones; see discussion in [32]).

Yet also certain other aspects differ between the modern forms and the long-necked fossils, such as the shape of the trunk. In modern larvae, the trunk appears well rounded, while in the fossils the trunk is slender, posteriorly tapering. Yet, other fossils have indicated that the ancestral condition for larvae of the group Myrmeleontiformia (of which Crocinae is an ingroup) was similar to the long-necked fossils [76], making it likely that the broader trunk evolved several times independently (see also further below). Therefore it is indeed likely that the long-necked fossils represent larvae of the group Crocinae (see also discussion in [33]).

### 4.2. Identity of the Other Specimens (0630–0633, 0901)

The other specimens resemble some larvae of Crocinae in the overall body shape. Yet, the fossils in addition have square-shaped heads. This aspect is shared with modern larvae of Nemopterinae, which is the sistergroup to Crocinae. The mandibles of most specimens are rather long in comparison to modern larvae of Crocinae. However, this can well be interpreted as a plesiomorphic trait still retained in early representatives of the group (see also discussion in [34]).

A different case is represented by the single small specimen with the prominent tooth with two cusps on each mandible (Figure 12). The detail of the tooth resembles that of a specimen also interpreted as a possible representative of Nemopterinae by Haug et al. [34]. It also strongly resembles the overall appearance of a stage 1 larva of Nemopterinae [60]. We therefore interpret also this specimen as a representative of Nemopterinae, most likely conspecific with the specimen with a similar tooth from Haug et al. [34].

### 4.3. Shape over Time

The dataset used here includes larval specimens from the Cretaceous and modern fauna, all supposedly of the group Nemopteridae. Currently, there is no available dataset for larvae of the Eocene or Miocene (which is available for other groups of Neuroptera [23]). The shape analyses are used for a comparative frame between the two available time slices.

**Figure 13:**
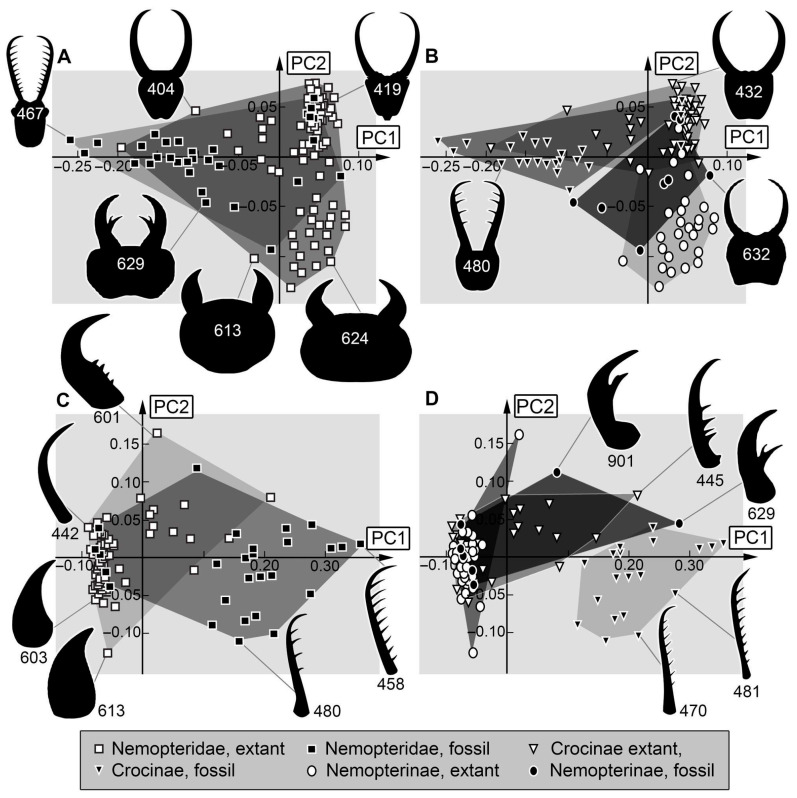
Scatterplots of PC1 vs. PC2 values. (**A**,**B**) Head + stylet shapes. (**A**) For Nemopteridae. (**B**) For Nemopterinae and Crocinae separately. (**C**,**D**) Stylet shapes. (**C**) For Nemopteridae. (**D**) For Nemopterinae and Crocinae separately. The polygons in different grey values mark the occupied areas of the compared groups in the morphospace.

*Head and stylet:* The morphospace occupation of the Cretaceous and the modern fauna are comparable in size, that of the modern fauna being a bit larger. Only three larvae of the Cretaceous lieoutside of the area that is occupied by extant larvae (Figure 13A). This is likely due to the influence of the stylets. Most modern-day larvae of Nemopteridae lack prominent teeth; it should not be surprising that the few larvae possessing prominent teeth (e.g., Specimen 467, Figure 13A) plot outside of the area of the modern larvae. In general, teeth-bearing larvae plot in the left half of the morphospace. The upper area is occupied by specimens with elongated, slender heads and stylets, while the lower area is occupied by specimens with broad heads and broad, short stylets.

There is a strong phylogenetic signal: the upper area is occupied by modern larvae of Crocinae, the lower one by modern larvae of Nemopterinae (Figure 13B). This highlights the morphological difference between these two groups. The separation of the fossils is less well expressed, as the teeth-bearing fossils likely representing larvae of Crocinae differ from the modern ones. Furthermore, the supposed larvae of Nemopterinae (e.g., specimens 629, 632) still possess a more plesiomorphic morphology, making them more similar to larvae of Crocinae than any of the modern larvae of Nemopterinae.

*Stylet:* The variability in the number and shape of teeth seems to be the main factor in the shape analysis (Figure 13C). The left half of the morphospace includes larvae without teeth, while the right half of the morphospace consists of teeth-bearing larvae. Most of the larvae that plot on the right side of the morphospace are larvae of the Cretaceous, while the left side is dominated by larvae of the modern fauna. Fossil larvae of Crocinae exclusively plot on the teeth-bearing half of the morphospace in an area that does not contain any other larvae than fossil larvae of Crocinae (Figure 13D). This indicates that fossil larvae had a quite different ecology than the modern larvae of Crocinae [33].

Similarly, some fossil larvae of Nemopterinae possess prominent teeth (e.g., specimens 629, 901; Figure 13D), leading to these plotting outside the areas of the modern larvae. Yet, the modern forms also occupy areas where no fossil plots, representing the short and stout stylets. Overall, the fossils show a slightly more diverse morphology of the stylets than their modern counterparts.

**Figure 14:**
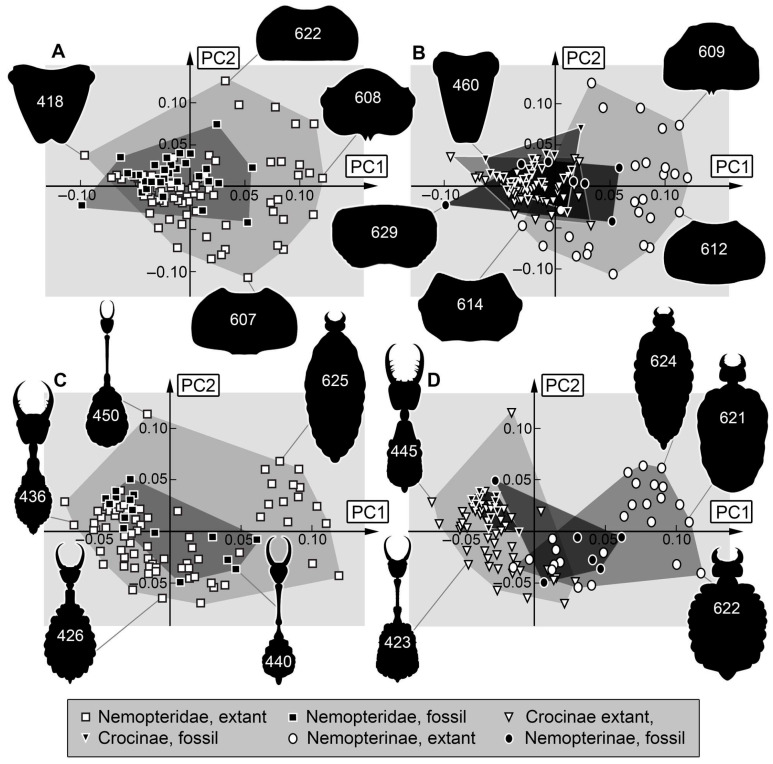
Scatterplots of PC1 vs. PC2 values. (**A**,**B**) Head capsule shapes. (**A**) For Nemopteridae. (**B**) For Nemopterinae and Crocinae separately. (**C**,**D**) Body + stylet shapes. For Nemopteridae. (**D**) For Nemopterinae and Crocinae separately. The polygons in different grey values mark the occupied areas of the compared groups in the morphospace.

*Head capsule*: Considering the head capsule without stylets (Figure 14A), the largest occupied area is represented by the modern fauna, specifically modern larvae of Nemopterinae (Figure 14B). This is different from the earlier study by Haug et al. [34].

**Figure 15:**
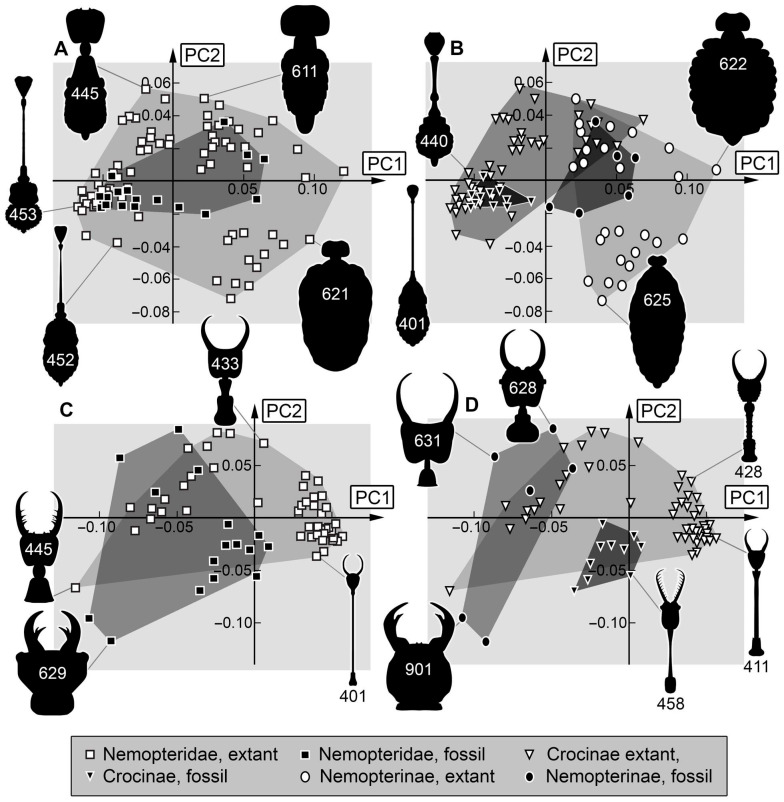
Scatterplots of PC1 vs. PC2 values. (**A**,**B**) Body without stylet shapes. (**A**) For Nemopteridae. (**B**) For Nemopterinae and Crocinae separately. (**C**,**D**) Head + prothorax + stylet shapes. (**C**) For Nemopteridae. (**D**) For Nemopterinae and Crocinae separately. The polygons in different grey values mark the occupied areas of the compared groups in the morphospace.

*Body shapes:* Body shapes, including head and stylets, but also without stylets, seem to be similar regarding the occupation of the morphospace. The occupation of the morphospace of the modern larvae is significantly larger than that of the Cretaceous larvae (Figure 14C and Figure 15A).

Yet, the sample size for the full body shape in fossil larvae is very small. Many larvae in amber do not have well-preserved trunks (e.g., Specimen 478, Figure 5A,B; Specimen 475, Figure 3). The sample size for extant larvae is much larger. Interestingly, the occupied area of fossil larvae of Crocinae is completely within that of extant larvae of Crocinae, whereas fossil larvae of Nemopterinae seem to plot outside of the area of extant larvae of Nemopterinae (Figure 14D and Figure 15B). This relates to the effects discussed above, especially the fossil larvae of Nemopterinae retaining many plesiomorphies.

**Figure 16:**
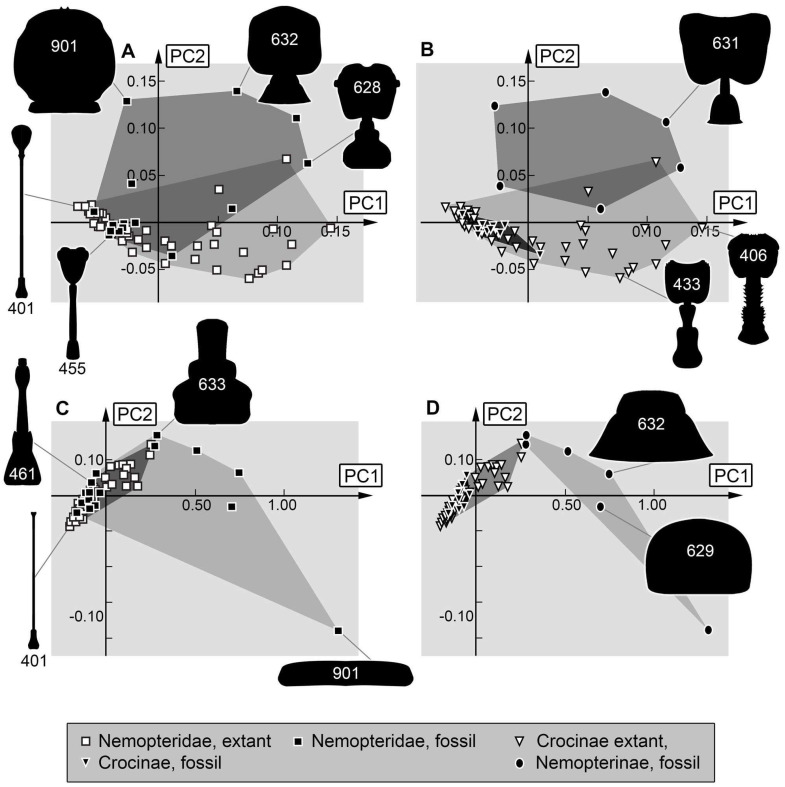
Scatterplots of PC1 vs. PC2 values. (**A**,**B**) Head + prothorax shapes. (**A**) For Nemopteridae. (**B**) For Nemopterinae and Crocinae separately. (**C**,**D**) Prothorax shapes. (**C**) For Nemopteridae. (**D**) For Nemopterinae and Crocinae separately. The polygons in different grey values mark the occupied areas of the compared groups in the morphospace.

*Head and prothorax shapes:* The overall signal of the here-expanded dataset of Cretaceous larvae partly occupying larger areas than the modern one is continued with the shapes of head and prothorax combined, in both cases with and without stylets (Figure 15C and Figure 16A). Since the head shape alone occupies a larger area of the morphospace for extant larvae, the shape of the prothorax can be assumed to be of morphological relevance. Yet, a major challenge is that there is no useful sample for extant larvae of Nemopterinae (Figure 15D and Figure 16B), as the posterior border of the prothorax is not well recognisable.

*Prothorax:* The observations in this analysis (Figure 16C) resemble those in the analyses of head and stylet. Yet, also here the non-availability of this aspect in modern larvae of Nemopterinae (Figure 16D) makes these results not informative.

**Figure 17:**
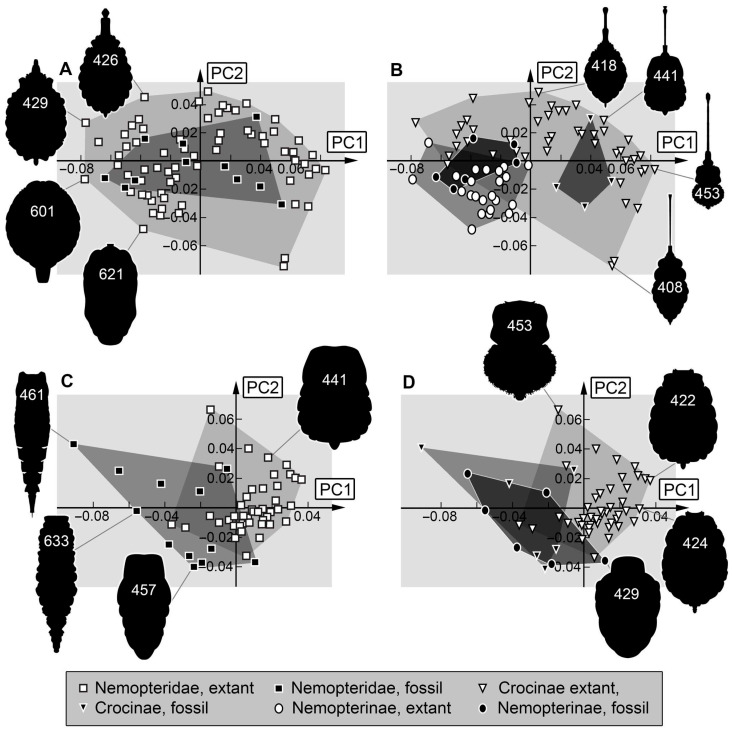
Scatterplots of PC1 vs. PC2 values. (**A**,**B**) Trunk shapes. (**A**) For Nemopteridae. (**B**) For Nemopterinae and Crocinae. (**C**,**D**) Trunk without prothorax shapes. (**C**) For Nemopteridae. (**D**) For Nemopterinae and Crocinae. The polygons in different grey values mark the occupied areas of the compared groups in the morphospace.

*Trunk:* As discussed earlier, the shape of the trunk is not well suited for shape analysis, as it is heavily dependent on the ontogenetic stage of the larva and how well the larva is fed [34]. Also, later stages are likely to have larger trunks, and for the modern fauna it is more common to report later larval stages, while amber seems to preferably preserve earlier stages. This may explain why the modern fauna is occupying a larger area than the Cretaceous one (Figure 17A). It is also apparent that the extant larvae of Nemopterinae and Crocinae seem to occupy different areas in the morphospace; this also applies to the fossil larvae within each group (Figure 17B).

*Trunk without prothorax:* The Cretaceous seems to occupy a slightly larger area of the morphospace than the modern fauna (Figure 17C). The trunk shape without prothorax reveals a principle difference between the modern larvae and the fossil ones. The left half of the morphospace is occupied by the slenderer fossil larvae, while the right half of the morphospace seems to be occupied by the broader extant larvae. This again emphasises the fact that myrmeleontiformian larvae had slenderer appearances in the Cretaceous [58,76,77,78].

### 4.4. Expanding of Morphospace by Adding New Specimens

**Figure 18:**
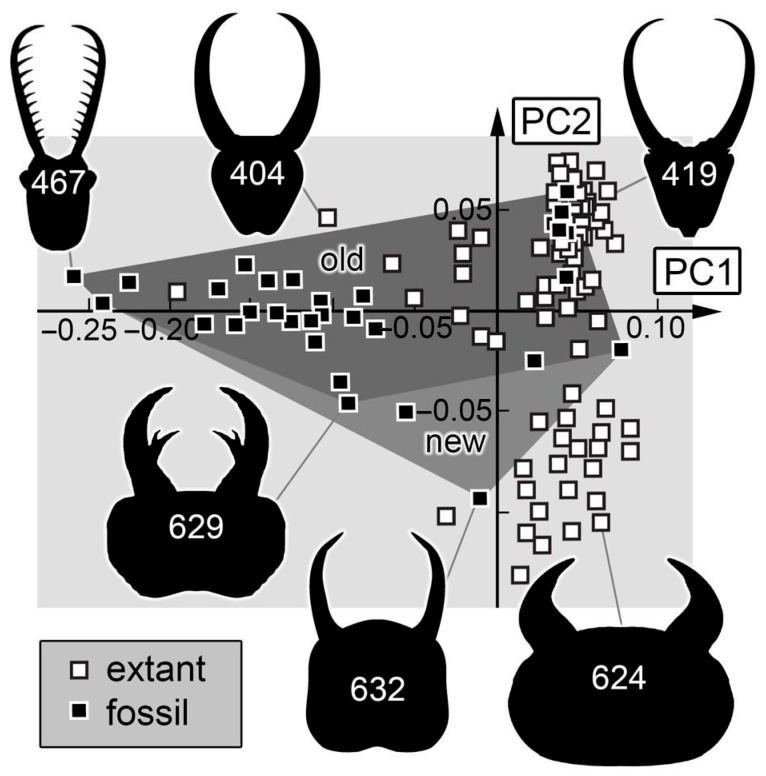
Scatterplot of PC1 vs. PC2 values of head + stylet shapes. Saturation plot comparing new fossil data (light grey) with previous dataset from Haug et al. [33,34] (dark grey).

The previous datasets by Haug et al. [33,34] were expanded by 17 new specimens, and 13 of them could be included in (at least some of) the analyses. Comparing the area occupied by previously known specimens with those including the new ones reveals an increase in diversity (Figure 18). In detail, this expansion is mostly due to a single specimen (632, Figure 18) that expands the morphospace into an area mostly occupied by modern larvae of Nemopterinae.

The same effect, that adding new specimens to an analysis still expands the morphospace, has been observed in other cases [79], including lacewing larvae [80,81]. This indicates that the datasets are still unsaturated and that not all morphologies present in the Cretaceous fauna have been recovered. This has been indicated by sample-size-corrected comparisons [23], in which the Cretaceous fauna of Nemopteridae was recovered as more diverse than the extant one. So far, the case still remains partly inconclusive for Nemopteridae, but it seems possible that also this group has undergone a decline since the Cretaceous.

### 4.5. Mosaic and Convergent Evolution

As pointed out above and in earlier studies [32,33,34], the fossil larvae of Nemopterinae and Crocinae differ from their modern counterparts in morphology and, coupled to that, presumably in ecology. Two major factors can be identified for these differences: mosaic evolution and convergent evolution.

Mosaic evolution, in this case, refers to the fact that not all autapomorphic characters known from modern representatives evolved at once. Instead, as to be expected, some new characters have evolved earlier and are observable in a fossil, while other characters appear still more plesiomorphic. Such differences are sometimes challenging to deal with from a taxonomic view but are to be expected in a phylogenetic one, see [82] vs. [83,84].

The other major factor, convergent evolution, leads to the phenomenon that two lineages independently evolve similar characters. Especially in closely related lineages, this is common, as in such cases the “starting conditions” are rather similar. Therefore, similar selective pressures lead to the evolution of similar morphologies [85].

When the first known fossil larvae of Nemopteridae were discussed [32], it remained open which characters were present due to convergence and which due to retaining plesiomorphies. In the meantime, the case has become much clearer. The slender trunk and the presence of prominent teeth in the stylets are known in numerous early representatives of Myrmeleontiformia [58,76,78,86]. The presence of these characters in the fossil larvae of Crocinae are plesiomorphies (see discussion in [76]). The long neck is a strong indicator that the fossils are indeed derivatives of the lineage towards modern representatives of Crocinae. The broad trunk in the modern forms evolved later, likely coupled to the flat digging behaviour [33]. The loss of teeth is likely coupled to a change in diet. This aspect needs to be further studied in modern larvae of which some still bear teeth in the first larval stage.

For the larvae of Nemopterinae, the broader trunk seems to have been present from rather early on, as the fossils already exhibit a comparatively broad one. The cervix is still quite apparent in the fossils but much more indistinct in the modern forms. Hence, it must have evolved later, convergently, for example, to larvae of owllions (larvae formerly attributed to the groups Ascalaphidae and Myrmeleontidae, status of both currently unclear [87]). Also, the shorter and stouter stylets were present to only a certain degree in the fossils; the stronger expressed ones of the modern larvae must have evolved later.

### 4.6. Convergent Evolution Is Not a Problem, but a Chance

Proponents of non-evolutionary concepts seem to like using cases of convergent evolution for “disproving” evolution (recent case for fossil larvae of Nemopteridae [88]). Yet, convergent evolution can in fact help to study aspects of evolution. Other than in experimental fields of biology, we cannot easily use parallel experimental set-ups for comparisons to study evolution. If several lineages evolved similar features convergently, we can see them as such parallel set-ups [89]. Especially concerning functional constraints and simple functional couplings to specific functions, convergent evolution can be informative [90]. In the case of Nemopteridae, we can recognise that the broader trunks in modern larvae of Crocinae and Nemopterinae are correlated to their digging behaviour. Still, the details of this behaviour are different, as modern larvae of Crocinae only dig themselves rather flat into the ground, basically only covering themselves with sand [33], while modern larvae of Nemopterinae dig rather deep [28].

Including fossils into quantitative comparisons has the potential to reliably recognise cases of convergence [91,92]. In some cases it will also be possible to resolve supposedly shared apomorphic characters as cases of convergence, opening the field for more finely graded evolutionary reconstructions [93].

## Data Availability

The original contributions presented in this study are included in the article/Supplementary Materials. Further inquiries can be directed to the corresponding author.

## Supplementary Materials

The following supporting information can be downloaded at https://www.mdpi.com/article/10.3390/insects17010011/s1, Figure S1: Possible larvae of Crocinae. A, B. Specimen 0472 (PED 0515). A. Ventral view. B. Dorsal view. C, D. Specimen 0477 (PED 2423). C. Ventral view. D. Dorsal view. E. Specimen 0473 (PED 0915). Table S1: Information about the specimens included in this study, especially in the shape analyses. File S1: Description of PC3 and following PCs. File S2: Detailed results of all shape analyses. Figure 1, Figure 2, Figure 3, Figure 4, Figure 5, Figure 6, Figure 7, Figure 8, Figure 9, Figure 10, Figure 11 and Figure 12 and Figure S1 in high resolution are available at https://doi.org/10.5281/zenodo.17822586 (accessed on 7 December 2025).
