# Supplementary material for: The Diversity of Spoon-Winged and Thread-Winged Lacewing Larvae Today and in Deep Time—An Expanded View
Source: Insects. 2025 Dec 20;17(1):11. doi: 10.3390/insects17010011 (PMC12842412; doi:10.3390/insects17010011)
Supplement: Supplementary file 1 [file insects-17-00011-s001.zip › Supplementary_Figure S1.pdf]

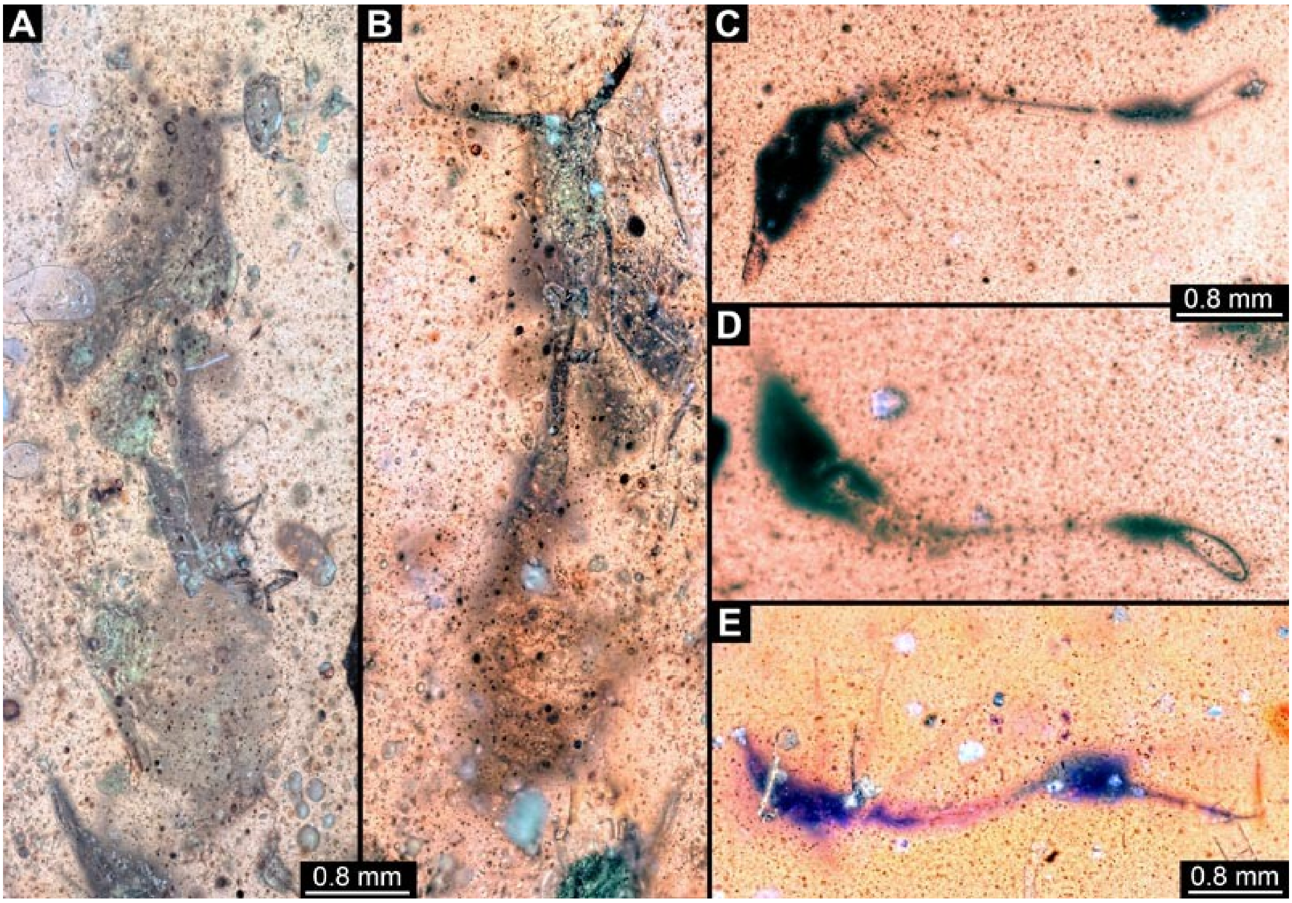

Supplementary Figure S1. Possible larvae of Crocinae. A, B. Specimen 0472 (PED 0515). A. Ventral view. B. Dorsal view. C, D. Specimen 0477 (PED 2423). C. Ventral view. D. Dorsal view. E. Specimen 0473 (PED 0915).

1) Specimen 0472 (PED 0515) is accessible in oblique view (Suppl. Fig. S1A, B) and largely concealed by dirt and gas bubbles. The stylets bear multiple teeth. The cervix is elongated and seems to be rather narrow and short in comparison to other specimens. The trunk end is not clearly visible. The preserved part of the specimen has a length of about 6.5 mm. This specimen was not included in the final analysis.

2) Specimen 0473 (PED 0915) is largely concealed by dirt and milky coating (Verlumung), therefore only the rough outline is apparent (Suppl. Fig. S1E). The specimen has a size of about 4.8 mm. This specimen was not included in the final analysis.

3) Specimen 0477 (PED 2423) is largely concealed by milky coating (Verlumung) in ventral (Fig. 2C) and dorsal view (Fig. 2D), only the rough outline is apparent. The specimen has a length of about 4.3 mm. This specimen was not included in the final analysis.
