# Supplementary material for: The Diversity of Spoon-Winged and Thread-Winged Lacewing Larvae Today and in Deep Time—An Expanded View
Source: Insects. 2025 Dec 20;17(1):11. doi: 10.3390/insects17010011 (PMC12842412; doi:10.3390/insects17010011)
Supplement: Supplementary file 1 [file insects-17-00011-s001.zip › Supplementary_File S1.pdf]

Supplementary File S1: Description of PC3 and following PCs

Analysis 1) Head and stylets:
PC3 explains 5.7% of the overall variation. Low values indicate a round head being narrower posteriorly and a broad anterior rim with rather straight, distally tapering stylets. High values indicate a rectangular head with curved stylets that are in the distal region as broad as in the proximal region.

PC4 explains 4.4% of the overall variation. It is dominated by the shape of the stylets. Low values indicate strongly curved, distally tapering stylets, while high values indicate rather straight stylets that are in the distal region as broad as in the proximal region.

PC5 explains 3.5% of the overall variation. It is dominated by the shape of the head. Low values indicate a rectangular head, while high values indicate a round head being narrower posteriorly.

PC6 explains 1.8% of the overall variation. It appears to be dominated by similar phenomena as PC4. Low values indicate rather straight stylets that are in the distal region as broad as in the proximal region, while high values indicate strongly curved, distally tapering stylets.

Analysis 2) Stylets:
PC3 explains 8.1% of the overall variation. It is dominated by the shape of the stylet tips. Low values indicate rounded stylet tips, while high values indicate tapering stylet tips.

PC4 explains 5.5% of the overall variation. It is dominated by the shape of the stylet tips. Low values indicate long, tapering stylet tips, while high values indicate rather short stylets and only slightly tapering stylet tips.

PC5 explains 2.6% of the overall variation. Low values indicate rather straight stylets in the proximal region with curved stylet tips. High values indicate curved stylets with rather straight stylet tips.

Analysis 3) Head capsule:
PC3 explains 12.9% of the overall variation. Low values indicate a deltoid shape of the head, while high values indicate a more rectangular head.

PC4 explains 9.3% of the overall variation. Low values indicate a rectangular head, while high values describe a round head.

PC5 explains 6.9% of the overall variation. It is dominated by the shape of the anterior rim of the head. Low values indicate a round head with a convex anterior rim. High values indicate a round head with a concave anterior rim.

PC6 explains 3.6% of the overall variation. It is dominated by the shape of the anterior rim of the head. Low values indicate a convex anterior rim, while high values indicate a concave anterior rim.

PC7 explains 2.4% of the overall variation. It appears to be dominated by similar phenomena as PC6. Low values indicate a convex anterior rim, while high values indicate a straight anterior rim.

Analysis 4) Body including stylets:
PC3 explains 11.9% of the overall variation. It is dominated by the shape of the head, yet also the shape of the stylets seems to influence this PC. It describes a short to long cervix with curved to straight stylets. Low values indicate a short, broad cervix and curved, tapering stylets. High values indicate a long, narrow cervix and rather straight stylets that are in the distal region as broad as in the proximal region.

PC4 explains 4.1% of the overall variation. It is dominated by the shape of the body. Low values indicate S-shaped lateral body rims with a tapering posterior end. High values indicate rather straight lateral body rims.

PC5 explains 3.0% of the overall variation. It is dominated by the shape of the head and the stylets. Low values indicate a head with straight lateral rims and straight stylets. High values indicate a concave lateral rim of the head and curved stylets.

PC6 explains 2.4% of the overall variation. It is dominated by the shape of the posterior trunk, yet the shape of the stylets also seems to influence this PC. Low values indicate a rather tapering posterior trunk and stylets that are in the distal region as broad as in the proximal region. High values indicate a rounded posterior trunk and tapering stylets.

PC7 explains 1.8% of the overall variation. It appears to be dominated by similar phenomena as PC6. Low values indicate a rather tapering posterior trunk with tapering stylets. High values indicate a rounded posterior trunk with stylets that are in the distal region as broad as in the proximal region.

Analysis 5) Body outline without stylets:
PC3 explains 8.0% of the overall variation. It is dominated by the shape of the head and the posterior rim of the body. Low values indicate an elongated head capsule with a stout cervix and a rounded posterior rim of the body. High values indicate a round head capsule with a narrow cervix and a tapering posterior rim of the body.

PC4 explains 5.1% of the overall variation. It is dominated by the shape of the head. Low values indicate a concave lateral rim of the cervix with an elongated head capsule. High values indicate a straight cervix with a round head capsule.

PC5 explains 3.0% of the overall variation. It is dominated by the shape of the head capsule. Low values indicate a small rectangular head capsule, while high values indicate an elliptical head capsule.

PC6 explains 2.5% of the overall variation. It is dominated by the shape of the head and the posterior end of the body. Low values indicate a round head capsule and a convex posterior end of the body. High values indicate an elongated head capsule and a concave posterior end of the body.

Analysis 6) Head, stylets, and prothorax:
PC3 explains 8.8% of the overall variation. It is dominated by the length of the cervix, yet also the shape of the stylets seems to influence this PC. Low values indicate a short cervix with stylets that are in the distal region as broad as in the proximal region. High values indicate a long cervix and tapering stylets.

PC4 explains 5.0% of the overall variation. It is dominated by the shape of the head capsule. Low values indicate a rather concave lateral rim of the head capsule, while high values indicate a convex lateral rim of the head capsule.

PC5 explains 4.1% of the overall variation. It appears to be dominated by similar phenomena as PC4. Low values indicate a convex lateral rim of the head capsule, while high values indicate a straight, broad head capsule.

PC6 explains 3.8% of the overall variation. It is dominated by the shape of the head capsule, yet the shape of the stylets also seems to influence this PC. Low values indicate a broad head capsule and strongly curved stylets. High values indicate a narrow head capsule and rather short, only slightly curved stylets.

PC7 explains 2.4% of the overall variation. It is dominated by the shape of the cervix and the length of the stylets. Low values indicate a cervix with a straight lateral rim and rather short stylets. High values indicate a concave lateral rim of the cervix and long stylets.

Analysis 7) Head and prothorax (no stylets):
PC3 explains 6.0% of the overall variation. It is dominated by the shape of the head. Low values indicate an elongated head capsule with a narrow cervix. High values indicate a rather short, round head capsule and a broad anterior rim of the cervix.

PC4 explains 4.3% of the overall variation. It is dominated by the shape of the head. Low values indicate a concave lateral rim of the head. High values indicate a straight cervix rim and a convex lateral rim of the head capsule.

PC5 explains 3.9% of the overall variation. It is dominated by the shape of the anterior rim of the head. Low values indicate a straight anterior rim of the head. High values indicate a convex anterior rim of the head.

PC6 explains 2.0% of the overall variation. It is dominated by the shape of the cervix, yet also the shape of the anterior rim of the head seems to influence this PC. Low values indicate a straight cervix with a rounded anterior rim of the head. High values indicate a concave lateral rim of the cervix.

Analysis 8) Prothorax:
PC3 explains 2.5% of the overall variation. It is dominated by the width of the anterior end. Low values indicate a rather broad anterior end, while high values indicate a rather slender anterior end.

PC4 explains 1.5% of the overall variation. It seems to be dominated by similar phenomena as PC3. Low values indicate a rather broad, rounded anterior end, while high values indicate a slightly tapering, slender anterior end.

PC5 explains 1.1% of the overall variation. It is dominated by the location of the widest part of the prothorax. Low values indicate a rather posteriorly located widest part of the prothorax, while high values indicate a rather anteriorly located widest part of the prothorax.

PC6 explains 0.57% of the overall variation. It is dominated by the shape of the anterior rim of the prothorax and the shape of the lateral rim. Low values indicate a rounded, rather broad anterior rim and a concave lateral rim, while high values indicate a rather narrow anterior rim and a rather straight lateral rim.

Analysis 9) Body outline without head capsule, i.e. trunk:
PC3 explains 7.0% of the overall variation. It is dominated by the shape of the thorax and the posterior end of the body. Low values indicate a concave lateral rim of the thorax and a convex posterior end of the body. High values indicate a rather straight lateral rim of the thorax and a concave posterior rim of the body.

PC4 explains 4.0% of the overall variation. It is dominated by the shape of the posterior trunk. Low values indicate a rounded convex posterior end with a fluent transition between thorax and posterior trunk. High values indicate a distinct transition between thorax and posterior trunk and a concave posterior rim of the body.

PC5 explains 3.0% of the overall variation. It is dominated by the shape of the anterior rim of the body. Low values indicate a tapering anterior rim, while high values indicate a broad rounded anterior rim.

PC6 explains 2.5% of the overall variation. Low values indicate a straight anterior and concave posterior rim of the body. High values indicate a concave anterior and convex posterior rim of the body.

PC7 explains 2.0% of the overall variation. It is dominated by the shape of the posterior end of the body, yet also the shape of the cervix seems to influence this PC. Low values indicate a convex posterior end of the body and a narrow cervix. High values indicate a concave posterior rim of the body and a broad cervix.

Analysis 10) Trunk outline without prothorax:
PC3 explains 15.6% of the overall variation. It is dominated by the anterior rim of the mesothorax. Low values indicate a broad rounded anterior rim of the mesothorax, while high values indicate a narrower anterior rim of the mesothorax.

PC4 explains 11.9% of the overall variation. It is dominated by the shape of the anterior rim of the mesothorax, yet also the shape of the trunk end seems to influence this PC. Low values indicate a broad anterior rim of the body with a clear distinction from the posterior trunk and an elongated trunk. High values indicate a rather curved anterior rim and a short, tapering trunk.

PC5 explains 5.5% of the overall variation. It appears to be dominated by the same phenomena as PC4. It is dominated by the shape of the anterior rim of the body, yet also the shape of the trunk end seems to influence this PC. Low values indicate a broad anterior rim of the mesothorax and a broader trunk end. High values indicate a rather curved anterior rim of the mesothorax and a narrower trunk end.

PC6 explains 4.3% of the overall variation. It is dominated by the shape of the posterior end of the body, yet also the position of the widest part of the body seems to influence this PC. Low values indicate a narrow, tapering, concave posterior end of the body and a further posteriorly located widest part of the body, while high values indicate a rounded, convex posterior end of the body and a further anteriorly located widest part of the body.

PC7 explains 2.3% of the overall variation. It is dominated by the shape of the thorax. Low values indicate a clear distinction between meso- and metathorax, while high values indicate a fluent transition between meso- and metathorax.

PC8 explains 1.9% of the overall variation. It is dominated by the shape of the anterior rim of the mesothorax. Low values indicate a convex anterior rim of the mesothorax, while high values indicate a concave anterior rim of the mesothorax.
