# Supplementary material for: The Diversity of Spoon-Winged and Thread-Winged Lacewing Larvae Today and in Deep Time—An Expanded View
Source: Insects. 2025 Dec 20;17(1):11. doi: 10.3390/insects17010011 (PMC12842412; doi:10.3390/insects17010011)
Supplement: Supplementary file 1 [file insects-17-00011-s001.zip › Supplementary_File S2.zip!/02/02.pdf]

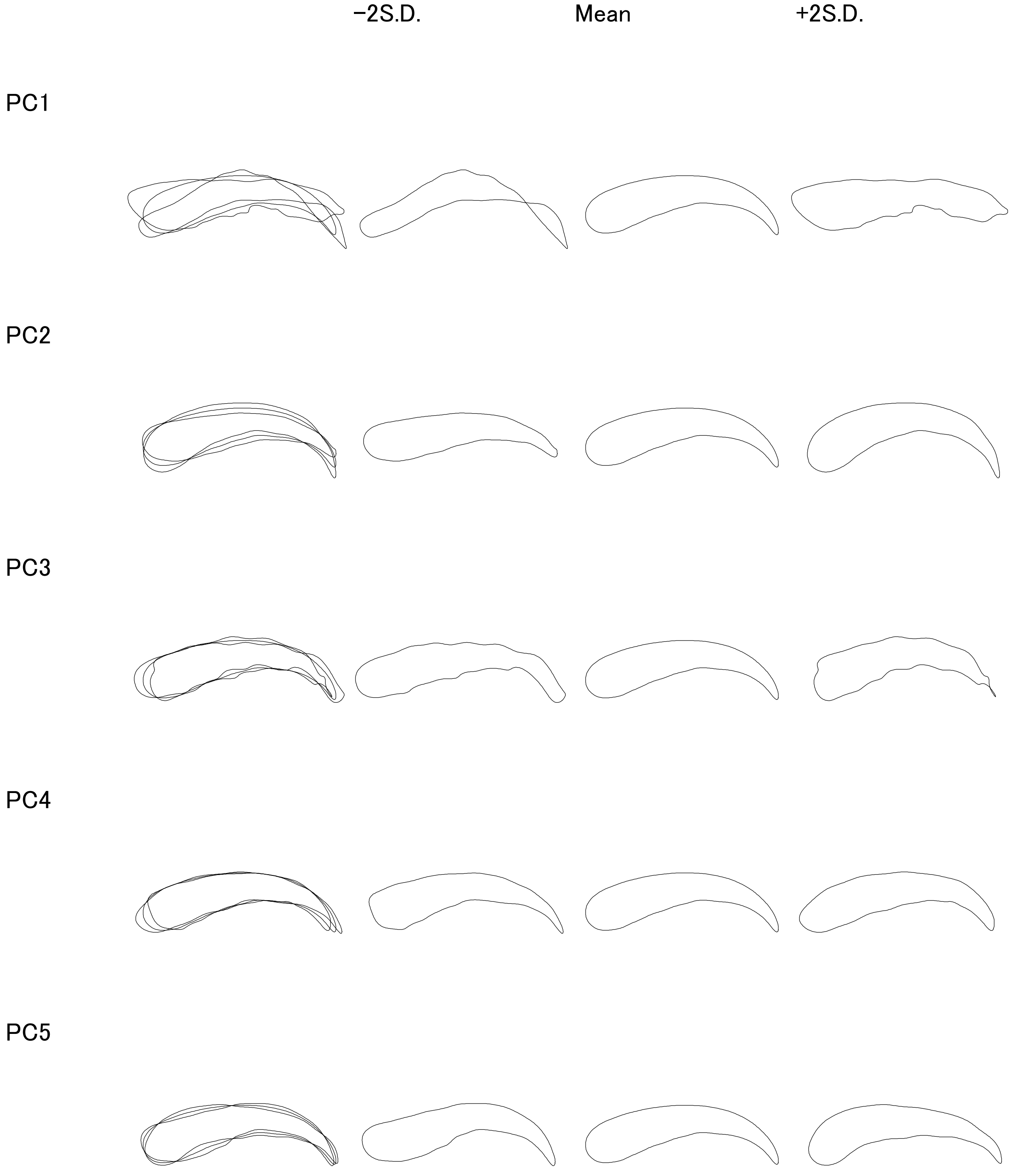
−2S.D.
Mean
+2S.D.
PC1
PC2
PC3
PC4
PC5
